# Antioxidant, Antiviral, and Anti-Inflammatory Activities of Lutein-Enriched Extract of *Tetraselmis* Species

**DOI:** 10.3390/md21070369

**Published:** 2023-06-21

**Authors:** Eun-A Kim, Nalae Kang, Seong-Young Heo, Jae-Young Oh, Seung-Hong Lee, Seon-Heui Cha, Won-Keun Kim, Soo-Jin Heo

**Affiliations:** 1 Jeju Bio Research Center, Korea Institute of Ocean Science and Technology (KIOST), Jeju 63349, Republic of Korea; euna0718@kiost.ac.kr (E.-A.K.); nalae@kiost.ac.kr (N.K.); syheo@kiost.ac.kr (S.-Y.H.); 2 Food Safety and Processing Research Division, National Institute of Fisheries Science, Busan 46083, Republic of Korea; ojy0724@korea.kr; 3 Department of Pharmaceutical Engineering, Soonchunhyang University, Asan 31538, Republic of Korea; seunghong0815@gmail.com; 4 Department of Marine Bio and Medical Sciences, Hanseo University, Seosan-si 31962, Republic of Korea; sunnycha@hanseo.ac.kr; 5 Department of Microbiology, College of Medicine, Hallym University, Chuncheon 24252, Republic of Korea; wkkim1061@hallym.ac.kr; 6 Department of Biology, University of Science and Technology (UST), Daejeon 34113, Republic of Korea

**Keywords:** *Tetraselmis* sp., antioxidant, antiviral, anti-inflammatory

## Abstract

Microalgae are proposed to have powerful applications for human health in the pharmaceutical and food industries. *Tetraselmis* species (sp.), which are green microalgae, were identified as a source of broad-spectrum health-promoting biological activities. However, the bioactivity of these species has not been elucidated. We aimed to confirm the antioxidant, antiviral, and anti-inflammatory effects of *Tetraselmis* sp. extract (TEE). TEE showed 2,2-diphenyl-1-picryl-hydrazyl-hydrate radical and hydrogen peroxide scavenging activities and reduced plaque formation in Vero E6 cells infected with vaccinia virus. TEE treatment also significantly inhibited nitric oxide (NO) production and improved cell viability in lipopolysaccharide (LPS)-induced RAW264.7 cells. These anti-inflammatory effects were further analyzed in LPS-induced RAW 264.7 cells and the zebrafish model. Further, TEE reduced induced NO synthase expression and proinflammatory cytokine release, including tumor necrosis factor-α, interleukin-6, and interleukin-1β, through MAPKs and NF-κB-dependent mechanisms. Further analysis revealed that TEE increased the survival rate and reduced cell death and NO production in an LPS-stimulated zebrafish model. Further, high-performance liquid chromatography revealed a strong presence of the carotenoid lutein in TEE. Overall, the results suggest that lutein-enriched TEE may be a potent antioxidant, antiviral, and anti-inflammatory agent that could be sustainably utilized in industrial applications.

## 1. Introduction

Microalgae are highly biodiverse and are among the most promising sources for new products and applications [1]. Microalgae are the fastest-growing plant cells in the world; hence, they are highly productive [2]. They are potential photoautotrophic microorganisms, with elevated contents and balanced compositions of several important and valuable secondary metabolites such as carotenoids, phycobilins, sterols, polyphenols, vitamins, fatty acids, and polysaccharides [1,3]. These metabolites are commonly identified in associated biological activities, having antioxidant, antidiabetic, antifungal, antiviral, anti-inflammatory, anticancer, neuroprotective, and immunomodulatory properties [3,4]. Therefore, microalgae have been proposed to have potent applications for human health in the pharmaceutical and food industries, as well as for decreasing the risk of illness [3,5]. Among the microalgae, *Tetraselmis* species, which are green marine microalgae, are commonly used in aquaculture because of their high nutritional value [6]. Studies have been conducted on this species to improve biomass and fatty acid productivity for biodiesel production and aquaculture fish oil utilization [7,8,9]. In addition, in a recent study, *Tetraselmis* sp. were identified as a potential source of a broad spectrum of health-promoting biological activities. Aqueous and ethanolic extracts of *Tetraselmis* sp. IMP3 and *Tetraselmis* sp. CTP4 have been studied for their antioxidant activity as measured by 2,2-diphenyl-1-picrylhydrazyl (DPPH), ferric reducing antioxidant power (FRAP), and ABTS methods and anti-inflammatory activity as measured by cyclooxygenase-2 (COX-2) inhibition [6]. Various organic extracts (methanol, ethyl acetate, chloroform, hexane, and acetone) of *Tetraselmis* sp. have been reported to exhibit antimicrobial activity against different human bacterial and fungal pathogens [10]. The methanol/dichloromethane extract of *Tetraselmis* sp. has been shown to exhibit antioxidant activity in DPPH, ABTS, ORAC, and TBARS assays [11,12]. *Tetraselmis* sp. have not yet been fully investigated for their bioactivity; therefore, further studies on biological activity and confirmation of biological mechanisms are necessary. In addition, a previous study by Lee et al. in 2021 showed that year-round *Tetraselmis* sp. can be cultivated in a semi-open raceway system (ORS) in Korea [9]. Therefore, it could be a useful biomass for industrial applications.

Inflammation is a complex biological response of tissues to harmful external stimuli such as pathogen invasion, tissue damage, and exposure to chemicals or endotoxins [13,14]. These stimuli lead to the uncontrolled release of nitric oxide (NO), prostaglandins (PGs), and proinflammatory cytokines such as tumor necrosis factor-alpha, interleukin (IL)-1β, and IL-6, which can cause various serious diseases such as cancer, diabetes, obesity, atherosclerosis, and neurological diseases [13,14,15]. In addition, free radicals can damage cells and cause severe diseases such as cancer, aging, diabetes, and cardiovascular and neurodegenerative disorders [16,17]. Antioxidants prevent and stabilize the damage caused by free radicals by supplying electrons from antioxidants to damaged cells [16,18]. Therefore, extensive research on anti-inflammatory agents and antioxidants for the prevention of health disorders is ongoing. Furthermore, coronavirus disease 2019 (COVID-19), declared a pandemic by the World Health Organization, has spread to almost all countries globally, posing an unprecedented threat to the global economy and human health [19,20,21]. Moreover, vaccinia virus (VACV) belonging to the Orthopoxvirus (OPV) genus and closely related to the variola virus, the causative agent of smallpox, was declared to be eradicated in 1979 [22,23]. However, with the increasing number of zoonoses, the development of strategies to combat OPV infections by introducing new therapeutic and preventive measures has become widely accepted [22]. To combat these crises, it is imperative to investigate potential antiviral materials that can be used effectively for their treatment. This study aimed to provide a broad overview of the biological activities of *Tetraselmis* sp. Among their potential antioxidant, antiviral, and anti-inflammatory activities, we first evaluated the anti-inflammatory effect of the ethanol extract of *Tetraselmis* sp. (TEE) by inhibiting inflammatory responses factors and verifying the biological mechanism in lipopolysaccharide (LPS)-stimulated RAW264.7 cells and a zebrafish model. To date, there are no reports explaining the plausible biological mechanisms and inflammatory response factors related to the anti-inflammatory effects of *Tetraselmis* sp. in vitro and in vivo. We also identified the active compounds in *Tetraselmis* sp. extract.

## 2. Results

### 2.1. Screening of Antioxidant, Antiviral, and Anti-Inflammatory Activities of TEE

**Figure 1:**
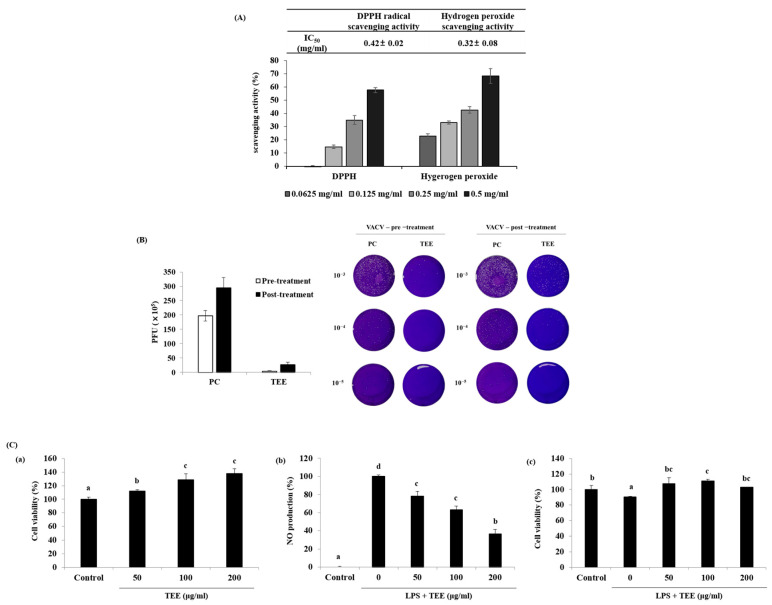
Antioxidant, antiviral, and anti-inflammatory activities of TEE. (**A**) Antioxidant activity was measured by determining the DPPH and hydrogen peroxide scavenging activities. (**B**) Antiviral activity was determined using the plaque assay for pre− and post−TEE treatment in Vero E6 cells infected with VACV for 24 h. (**C**) Anti-inflammatory activity was analyzed for (**a**) toxicity, (**b**) cell viability, and (**c**) NO production using the MTT and Griess assays. The data are expressed as the mean ± standard deviation of triplicate experiments. ^a–d^ Values with superscript letters differed significantly (*p* < 0.05, by Duncan’s multiple rages test).

The approximate chemical compositions of *Tetraselmis* sp. moisture, carbohydrate, crude protein, crude lipid, and ash were 11.87%, 19.81%, 34.74%, 6.85%, and 26.7%, respectively, according to the AOAC methods (Table 1). The TEE extract yield was 41.68%. We screened for the multifunctional activity of TEE, including its antioxidant, antiviral, and anti-inflammatory activities. We first identified the DPPH and hydrogen peroxide scavenging activities of TEE to determine its potential antioxidant activity. As shown in Figure 1A, TEE significantly increased the scavenging activity of DPPH radical and hydrogen peroxide with IC_50_ values of 0.42 mg/mL and 0.32 mg/mL, respectively. Next, we performed plaque assays for viral titer to confirm the antiviral activity of TEE. We investigated whether pre-treatment and post-treatment with TEE following infection reduced plaque formation. Both treatment methods inhibited plaque formation in VACV-infected Vero E6 cells. To further confirm the anti-inflammatory activity, we assessed toxicity, NO production, and cell viability in RAW 264.7 cells. As depicted in Figure 1C(a), TEE did not show cytotoxicity at concentrations of 50–200 μg/mL in RAW264.7 cells following the MTT assay. Treatment with LPS evidently increased NO production compared to the control group (Figure 1C(b)). Moreover, TEE showed particularly reduced NO levels relative to the LPS group (21.68%, 36.73%, and 63.52%, respectively) and increased cell viability at 50, 100, and 200 μg/mL (Figure 1C(b,c)). Taken together, TEE showed antioxidant, antiviral, and anti-inflammatory activities. In this study, we first focused on the anti-inflammatory properties of TEE, and through follow-up experiments, we confirmed its inhibition of inflammatory factors, elucidated the underlying biological mechanism in LPS-induced RAW264.7, and checked the inhibitory efficacy of NO production in LPS-stimulated zebrafish.

### 2.2. Anti-Inflammatory Effects of TEE on the Expression of iNOS and COX-2 Protein and Production of Proinflammatory Cytokines in LPS-Stimulated RAW 264.7 Cells

**Figure 2:**
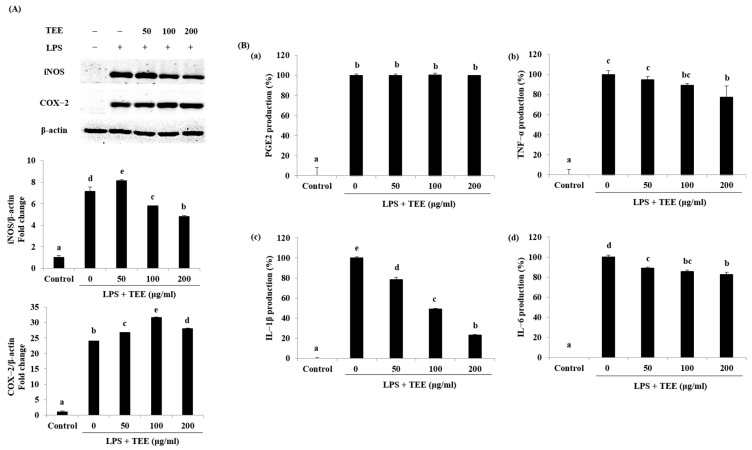
Inhibition effect of TEE on LPS-stimulated iNOS, COX−2 protein, and proinflammatory cytokines in RAW264.7 cells. (**A**) Cell lysates were extracted, and iNOS and COX−2 protein levels were analyzed using Western blotting. ImageJ software (version 1.46r) used was normalized to β-actin. (**B**) The supernatants were collected and analyzed for (**a**) PGE2, (**b**) TNF−α, (**c**) IL−1β, and (**d**) IL−6 production using ELISA. The data are expressed as the mean ± standard deviation of triplicate experiments. ^a–e^ Values with superscript letters differed significantly (*p* < 0.05, by Duncan’s multiple rages test).

The inhibition of inflammatory response factors such as iNOS, COX-2, and proinflammatory cytokines was investigated by Western blotting and ELISA. As shown in Figure 2A, treatment with LPS sharply improved the protein levels of iNOS and COX-2 compared to the control group. However, TEE significantly suppressed iNOS expression at concentrations of 100 and 200 μg/mL. The expression of COX-2 protein was not altered following TEE treatment. As shown in Figure 2B, TEE inhibited the production of proinflammatory cytokines including PGE2, TNF-α, IL-1β, and IL-6. TEE did not affect PGE2 levels. However, TEE inhibited the production of IL-1β by 21.71%, 50.96%, and 76.98% in a concentration-dependent manner. The inhibition rates of TNF-α and IL-6 at 200 μg/mL were 22.70% and 17.57%, respectively. TEE suppressed the expression of iNOS and production of proinflammatory cytokines, including TNF-α, IL-1β, and IL-6.

### 2.3. Effect of TEE on MAPKs and NF-κB Signaling Pathways in LPS-Stimulated RAW 264.7 Cells

**Figure 3:**
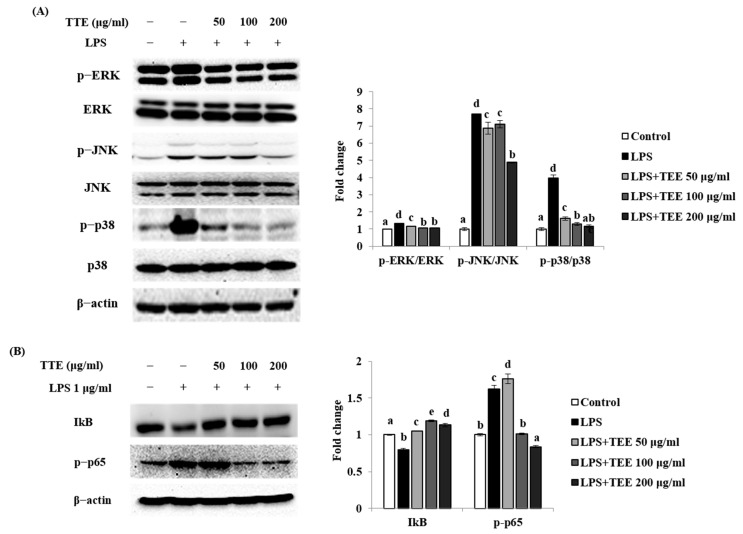
Inhibition effect of TEE on LPS-induced MAPKs and NF−kB signaling pathway in RAW264.7 cells. (**A**) Cell lysates were extracted, and protein levels of (**A**) MAPKs (p−ERK, ERK, p-JNK, JNK, p−p38, p−38) and (**B**) NF-κB (IkB, p−p65) were analyzed using Western blotting. ImageJ software (version 1.46r) used was normalized to β-actin. The data are expressed as the mean ± standard deviation of triplicate experiments. ^a–e^ Values with superscript letters differed significantly (*p* < 0.05, by Duncan’s multiple rages test).

To determine the intracellular signaling pathways underlying the suppressive effect of TEE on inflammatory response factors, we investigated the MAPKs and NF-κB signaling pathways by Western blotting. As shown in Figure 3A, treatment with LPS markedly upregulated ERK, JNK, and p38 phosphorylation compared to those in the control groups. Treatment with TEE selectively downregulated JNK and p38 phosphorylation. However, ERK phosphorylation was unaffected by TEE treatment. Treatment with LPS considerably increased p65 phosphorylation and decreased degranulation of IkB in the cytosol compared with the control group (Figure 3B). In contrast, TEE treatment suppressed p65 phosphorylation and increased IkB levels. Therefore, TEE induced degranulation of IkB, and this phosphorylation upregulated p65 translocation from the cytosol to the nucleus. Thus, we concluded that TEE inhibited inflammatory response factors through the JNK, p38 MAPK, and NF-κB signaling pathways in LPS-activated macrophages.

### 2.4. Effect of TEE on Cell Death and Production of NO in LPS-Treated Zebrafish

**Figure 4:**
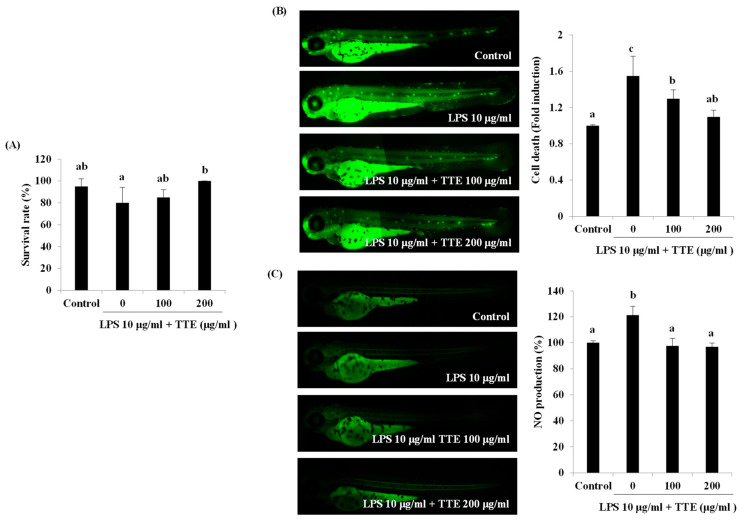
Inhibitory effect of TEE on LPS-stimulated cell death and NO production in a zebrafish model. (**A**) Survival rate was evaluated by counting the number of surviving zebrafishes at 7 dpf. (**B**) Cell death was analyzed using acridine orange staining. (**C**) NO production was measured using DAF-FM-DA staining. Quantitative analysis of fluorescence was performed using ImageJ software (version 1.46r). The data are expressed as the mean ± standard deviation of triplicate experiments. ^a–c^ Values with superscript letters differed significantly (*p* < 0.05, by Duncan’s multiple rages test).

We further investigated whether TEE exerts anti-inflammatory activity in in vivo LPS-stimulated zebrafish. As shown in Figure 4A, the survival rate that was reduced following treatment with LPS was increased with TEE treatment. Moreover, LPS considerably increased cell death by approximately 1.55-fold compared to the control group, which was subjected to acridine orange staining (Figure 4B). However, TEE treatment inhibited cell death by approximately 0.25- and 0.45-fold compared to LPS treatment at concentrations of 100 and 200 μg/mL, respectively. To investigate the effect of TEE on NO production, we evaluated NO generation using DAF-FM-DA staining (Figure 4C). LPS treatment increased NO production compared to that observed in the control group. However, TEE treatment decreased LPS-induced NO generation by 23.78% and 24.55% at 100 and 200 μg/mL, respectively.

### 2.5. Identification of Lutein from TEE

**Figure 5:**
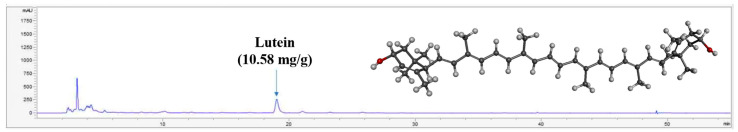
Lutein content in TEE following HPLC analysis.

To determine the TEE components, we conducted HPLC chromatogram analysis of TEE. Microalgae species have been studied for their production of various carotenoids [24]; therefore, to identify the active components of TEE, we mainly focused on carotenoids. Figure 5 depicts an HPLC chromatogram of TEE at a wave length of 454 nm. The detected characteristic was attributed to lutein (10.58 mg/g). Therefore, we confirmed that lutein is a prominent component of TEE. The calibration curve for lutein is shown in Supplementary Figure S1.

## 3. Discussion

Microalgae have valuable advantages, such as rapid growth, high photosynthetic efficiency, and the possibility of cultivation under production conditions; however, they are less studied than macroalgae/seaweed [25]. A previous study by Lee et al. in 2021 indicated that the biomass production of *Tetraselmis* sp. was 32.14 g/m^2^/d in semi-ORP under Korean climate conditions [9]. To utilize *Tetraselmis* sp. as a therapeutic agent, we investigated the antioxidant, antiviral, and anti-inflammatory effects of TEE.

Measurement of DPPH radical scavenging activity is the first approach for evaluating the potential antioxidant properties of a molecule. Hydrogen peroxide is one of the major types of reactive oxygen species (ROS), and determination of its scavenging activity is widely used and is the convenient method to confirm antioxidant activity [26,27]. Therefore, these two methods were selected for our study. As a result, TEE showed 0.42 mg/mL and 0.32 mg/mL DPPH radical and hydrogen peroxide scavenging activities, respectively. Previous studies have shown DPPH radical scavenging activity in hexane, ether, and methanol/dichloromethane extracts of *Tetraselmis* sp., which exhibited IC_50_ values of 2.4, 2.5, and 0.8 mg/mL, respectively [1,11]. TEE showed higher radical scavenging activity than the aforementioned extracts, making it a potential antioxidant source. Next, we measured the antiviral activity of TEE against vaccinia virus using a plaque assay. The results of this assay indicated that both pre- and post-treatment with TEE suppressed VACV plaque formation. Previous studies have shown the antiviral activity of a number of bioactive compounds, including lectins, polysaccharides, carotenoids, and peptides derived from microalgae, against some common human and animal viruses [28,29,30]. However, research on the antiviral effect of *Tetraselmis* sp. has not been confirmed, thus warranting further investigation of these species as candidate microalgae with antiviral activity. Furthermore, in both the LPS-stimulated RAW264.7 cells and the zebrafish model, TEE suppressed NO production in a concentration-dependent manner and did not show toxicity at concentrations below 200 μg/mL. NO is a free radical that has complex roles in both health and disease and is synthesized by various synthases, viz. iNOS, neuronal nitric oxide synthase (nNOS), and endothelial nitric oxide synthase (eNOS) [14]. iNOS is the key enzyme that produces NO in response to stimulation by LPS or other proinflammatory mediators [14,15].

Zebrafish are a very popular in vivo model used in genetic, toxicological, and pharmacological studies owing to their multiple advantages, such as extrauterine development, high fecundity, and similarity to human tissues and genomes [13,14]. Owing to this, the LPS-stimulated zebrafish model has been used to evaluate anti-inflammatory materials that can be used as drugs and/or health functional foods [13,14]. In this study, TEE effectively reduced NO production both in vitro and in vivo. Thus, TEE was confirmed to have a potent anti-inflammatory effect. In addition, TEE reduced the protein levels of iNOS and the proinflammatory cytokine production levels of TNF-α, IL-1β, and IL-6 in LPS-induced RAW264.7 cells based on Western blot and ELISA results. The iNOS protein triggers inflammation, and cytokines such as TNF-α, IL-1β, and IL-6 are important pathogenic factors in various inflammatory disorders [15,31]. Reduction of these mediators is considered a promising therapeutic approach for the clinical treatment of inflammatory diseases [32]. According to these results, TEE may be effective for the prevention and treatment of inflammatory diseases by suppressing proinflammatory cytokines. MAPKs and NF-κB are key signaling pathways for exploring the mechanisms of inflammatory responses that regulate the expression of the proinflammatory cytokines iNOS and COX-2 in LPS-stimulated macrophages [13,32]. Treatment of LPS-induced RAW264.7 cells with TEE reduced the phosphorylation of MAPKs such as ERK, JNK, and p38, as well as NF-κB p-65 levels and the degradation of IkB levels in the cytosol. These results confirmed that TEE is an effective anti-inflammatory inhibitor of proinflammatory mediators and cytokines that acts by suppressing the MAPKs and NF-κB signaling pathways. Further, *Tetraselmis* sp. have nutritional value in the form of proteins, lipids, minerals, carotenoids, and phenolic compounds [33]. The anti-inflammatory active components of TEE were confirmed through HPLC. TEE contained lutein (10.58 mg/g, 4.41 mg/g Dry weight (DW)). Lutein, a xanthophyll, is a lipid-soluble primary carotenoid that humans obtain from their diet and is known to have antioxidant and anti-inflammatory health benefits in preventing heart attack, metabolic syndromes, and macular degenerative disease [15,34,35]. The global market for carotenoids was estimated to be worth USD 1.5 billion in the year 2020 and is projected to reach USD 2.2 billion by 2027, with lutein estimated to account for approximately 15–20% of the total market [34,36]. However, as biological sources are still limited and expensive, this enormous global carotenoid market is now firmly dominated by chemical synthesis. Therefore, the natural lutein market is attracting attention for use in the pharmaceutical, dietary supplement, food, animal, and fish feed industries [37]. In previous studies, the lutein content of *Tetraselmis* sp. CTP4, *Tetraselmis* chui, and *Tetraselmis* sp. M8 were found to be 3.17, 0.62, and 0.66 mg/g DW, respectively, while vegetables such as kale, broccoli, and cilantro showed contents of 0.03, 0.04, and 0.08 mg/g [3,34,36,38]. In this study, TEE, as a potential lutein producer, showed anti-inflammatory, antioxidant, and antiviral activities; however, further research is needed to identify additional efficacy-related factors and mechanisms that can prove the antioxidant and antiviral properties.

## 4. Materials and Methods

### 4.1. Algal Cultivation and Extraction

The green microalga *Tetraselmis* sp. MBEyh04Gc (KCTC 12432BP) was obtained from the Marine Bioenergy R&D Consortium (MBE) of Inha University, Incheon, Korea. *Tetraselmis* sp. were cultivated as previously described by Lee et al. in 2021 [9]. A total of 10g of powdered *Tetraselmis* sp. was dissolved using 70% EtOH with sonication for 1 h, three times, and then filtered. Finally, *Tetraselmis* sp. EtOH extract (TEE) was obtained by rotary evaporation and freeze-dried. The yield was calculated by subtracting the dried weight of the residue from 1 mL of dried TEE and is expressed as a percentage.

### 4.2. Proximate Composition of Tetraselmis sp.

The proximate composition of *Tetraselmis* sp. was determined following methods described by the Association of Official Analytical Chemists (AOAC) [39]. Crude protein content was confirmed using the Kjeldahl method, and crude lipid content was determined using the Soxhlet method. Moisture was prepared by keeping the sample in a dry oven, and crude ash was dried in a dry-type furnace at 550 °C.

### 4.3. Determination of Antioxidant Activity

#### DPPH Radical and Hydrogen Peroxide Scavenging Assay

To estimate the antioxidant activity of TEE, DPPH radical and hydrogen peroxide scavenging activities were determined according to previously reported methods [40,41]. Briefly, a solution of 4 × 10^4^ M DPPH in methanol was prepared, and 100 µL of this solution was mixed with 100 µL of TEE in multiple concentrations. The mixtures were incubated in a shaking incubator for 30 min at 25–30 °C. After incubation, the absorbance was measured spectrophotometrically at 517 nm (Synergy HT Multi-Detection microplate reader, BioTek, Winooski, VT, USA). For hydrogen peroxide scavenging activity, 100 µL of TEE in multiple concentrations and 0.1 M phosphate buffer (pH 5.0) was mixed with 10 mM hydrogen peroxide solution and incubated for 5 min at 37 °C. Then, 30 μL of 1.25 mM ABTS and 1 unit/mL peroxidase were added. The mixtures were incubated for 10 min at 37 °C, and the incubated product was measured at 405 nm using a microplate reader (Synergy HT Multi-Detection microplate reader, BioTek, Shoreline, DC, USA).

### 4.4. Determination of Antiviral Activity

#### Plaque Assay

African green monkey kidney Vero E6 cells were purchased from the American Type Culture Collection (CRL-1586, Washington, DC, NW, USA) and were cultured in DMEM (Dulbecco’s Modified Eagle’s Medium, Gibco, Billings, MT, USA) supplemented with 10% fetal bovine serum (FBS, Gibco, USA) and 1% penicillin-streptomycin (Thermo Fisher Scientific, Waltham, MA, USA). They were maintained at 37 °C in an incubator in a 5% CO_2_ atmosphere. The cells were essential to subculture every 3 to 4 days at 70–80% confluence. The vaccinia virus was supplied by the National Culture Collection for Pathogens (Cheongju, Republic of Korea). The virus stock (1 × 10^5^ virus/mL) was titrated using a plaque assay and stored at −80 °C. Antiviral effects of TEE were tested via a plaque assay using two treatments: pre-sample treatment and post-sample treatment. Vero E6 cells were seeded in a 6-well plate at a density of 1 × 10^6^ cells per well and incubated in 10% FBS-supplemented DMEM until the wells were filled with a monolayer of cells (100% confluency). The cells were washed twice with 1 mL of 1 X phosphate buffered saline (PBS, WELGEN, Gyeongsan, Republic of Korea) and 0.25 mL of DMEM, and 0.5 mL of each sample/PBS treated cell media dilution was added to the respective cells on the wall of the well. The cells were incubated at 37 °C in an incubator in a 5% CO_2_ atmosphere for 90 min, with gentle shaking every 15 min to allow for virus adsorption. After adsorption, the inoculum was removed from the cells, and 2 mL of 2% FBS-supplemented DMEM was added to the cells and incubated at 37 °C in an incubator in a 5% CO_2_ atmosphere for 2–3 days. The medium was then removed from the cells, and 1 mL of 0.1% crystal violet was added to the cells for staining for 30 min at room temperature. The crystal violet solution was discarded, and the cells were washed with PBS and dried. Virus titers were calculated by counting the number of plaques, using the following formula:

PFU/mL = Number of plaques/(dilution factor × volume of diluted virus/well)

where PFU = plaque forming unit.

### 4.5. Determination of Anti-Inflammatory Activity

#### 4.5.1. MTT and NO Production Assay

Murine macrophage RAW264.7 cells were obtained from the Korean Cell Line Bank (KCLB NO 40071, Seoul, Republic of Korea) and were cultured in DMEM supplemented with 10% FBS and 1% penicillin and streptomycin. Cells were maintained in a controlled environment at 37 °C in an incubator in a 5% CO_2_ atmosphere. Subculture of RAW264.7 cells was performed once every 2 days. The cells were seeded in 24-well plates at a concentration of 1.5 × 10^5^ cells/mL and incubated for 16 h. Cells were treated with TEE in multiple concentrations for 1 h. Then, lipopolysaccharide (LPS from Escherichia coli O111:B, Sigma-Aldrich, St. Louis, MO, USA; 1 µg/mL) was added for co-treatment for another 24 h of incubation. The cytotoxicity and cell viability of TEE were assessed using the 3-(4,5-Dimethyl-2-thiazolyl)-2,5-diphenyltetrazolium Bromide (MTT) assay, and NO production was evaluated using the Griess assay [32].

#### 4.5.2. PGE_2_ and Proinflammatory Cytokines Production Assay

The procedure used to estimate the anti-inflammatory activity, also relevant to this assay, is explained in Section 4.5.1. The PGE_2_ and proinflammatory cytokines (TNF-α, IL-6, IL-1β) of the culture supernatant were measured using a mouse ELISA kit (R&D Systems, Minneapolis, MN, USA) and a mouse ELISA kit (Biosciences, Franklin Lakes, NJ, USA) following the manufacturer’s instructions.

#### 4.5.3. Western Blots

The protein expression levels of iNOS, COX-2, p-ERK, ERK, p-JNK, JNK, p-p38, p38, Ik-B, p-p65, and β-actin were analyzed by Western blotting. The cells were seeded as described above, treated with samples, and treated with LPS for 24 h or 15 min following the method by Thilina et al. [42]. Protein concentrations of the cell lysates were measured using a BCA^TM^ protein assay kit (Thermo Scientific, Waltham, MA, USA) according to the manufacturer’s instructions. Blocked membranes (3% bovine serum albumin and 2% skim milk) were incubated with primary (1:1000 dilution, Cell Signaling Technology, Danvers, MA, USA) and goat anti-mouse or anti-rabbit secondary (1:3000 dilution, Santa Cruz Biotechnology, Dallas, TX, USA) antibodies. The protein bands were detected using FUSION SOLO (Vilber Lourmat, Marne La Vallée, France), and the intensity quantification of the Western blot results was measured using ImageJ software (version 1.46r). [43].

#### 4.5.4. Breeding of Zebrafish and NO Production Assay

Zebrafish were maintained in 3.5 L acrylic tanks under the following conditions: at 28.5 ± 1 °C, with a 14/10 h light/dark cycle, and they were fed twice daily (Tetra GmbH, Melle, Germany). The day before, the zebrafish were interbred (breeding one female and two males), spawning was stimulated by the onset of light. Embryos were transferred from natural spawning to Petri dishes containing media [44]. At 7–9 h post-fertilization (hpf), the embryos were transferred to 12-well plates (15 embryos/well) and were first incubated for 1 h with selected TEE concentrations (100 and 200 μg/mL) and then sensitized with LPS for 3 days post-fertilization (dpf). At 3 dpf, zebrafish larvae were stained with DAF-FM-DA solution (Sigma-Aldrich, USA) or acridine orange solution (Sigma-Aldrich, USA) for the detection of NO production and determination of cell death following the method by Kim et al. [32]. Survival rate was measured at 7 dpf using a DS-Fi1c digital camera (Nikon, Tokyo, Japan). Fluorescence intensity was quantified using the ImageJ program (version 1.46r). The zebrafish experiments were performed in accordance with the regulation of the Animal Care and Use Committee of the Animal Center of Korea Institute of Ocean Science and Technology (November 2021).

### 4.6. HPLC Chromatogram Analysis

TEE (30 mg/mL) was dissolved in chloroform:methanol (50:50, *v*/*v*) solution and filtered through a 0.45 μm PVDF membrane filter. TEE was analyzed using an Agilent 1260 Infinity II Quaternary Pump and DAD WR detector (Santa Clara, CA, USA). The analytical conditions of the HPLC system were as follows: column, YMC carotenoid C30 (4.6 × 250 mm, 3 μm); solvent system, water:methanol (25:75, *v*/*v*) and ethyl acetate; gradient program, 83:17 *v*/*v* at 0 min, 70:30 *v*/*v* at 10 min, 70:30 *v*/*v* at 25 min, 20:80 *v*/*v* at 30 min, 0:100 *v*/*v* at 35 min, 0:100 *v*/*v* at 40 min, 83:17 *v*/*v* at 50 min, 83:17 at 55 min; flow rate, 1 mL/min; temperature, room temperature; injection volume, 10 μL; detector wavelength, 454 nm. The carotenoid contents of TEE were quantified by comparing the retention times and peak areas with the corresponding standard curve.

### 4.7. Statistical Analysis

All data were obtained in triplicates and are reported as the mean ± standard deviation (SD). Statistical analysis for comparing the data was performed using the IBM SPSS Statistics (version 20) software using one-way ANOVA, followed by Duncan’s multiple range test. Statistical significance was set at *p* values < 0.01.

## 5. Conclusions

In the present study, the antioxidant and antiviral effects of a lutein-enriched extract of *Tetraselmis* sp. were investigated using the DPPH radical and hydrogen peroxide scavenging activities and the plaque formation inhibition of VACV, respectively. In particular, we demonstrated the anti-inflammatory activity through the suppression of NO and proinflammatory cytokine production by MAPKs and NF-kB signaling pathways in LPS-stimulated RAW264.7 cells and the inhibition of NO production in an LPS-induced zebrafish model. These microalgae could be cultivated in a 240,000 L semi-ORS, thus reducing the cost of production and increasing its availability and granting it the potential to be used as a functional agent in various industrial applications sustainably.

## Figures and Tables

**Table 1:** Proximate composition of *Tetraselmis* sp.

Composition	Moisture	Carbohydrate	Crude Protein	Crude Lipid	Ash
Content (%)	11.87	19.81	34.74	6.85	26.7

## Data Availability

The data presented in this study are available upon request from the corresponding author.

## Supplementary Materials

The following supporting information can be downloaded at https://www.mdpi.com/article/10.3390/md21070369/s1. Figure S1: The standard chromatogram of lutein. (A) Calibration curve of lutein. (B) HPLC chromatogram of standard lutein.
