# Supplementary material for: Antioxidant, Antiviral, and Anti-Inflammatory Activities of Lutein-Enriched Extract of Tetraselmis Species
Source: Mar Drugs. 2023 Jun 21;21(7):369. doi: 10.3390/md21070369 (PMC10381658; doi:10.3390/md21070369)
Supplement: Supplementary file 1 [file marinedrugs-21-00369-s001.zip › marinedrugs-2432577-supplementary.pdf]

Supplementary materials

(A)

(B)

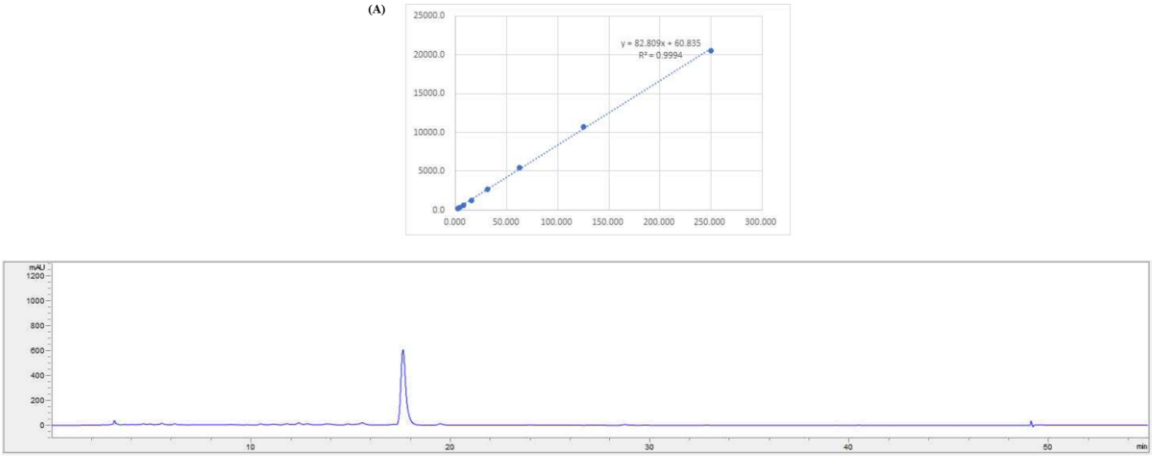

Figure S1. The standard chromatogram of lutein. (A) Calibration curve of lutein. (B) HPLC chromatogram of standard lutein
